# Elemental analysis of *Fadogia ancylantha* leaves used as a nutraceutical in Mashonaland West Province, Zimbabwe

**DOI:** 10.1515/biol-2022-0695

**Published:** 2023-09-30

**Authors:** Tom Muzenda, Ryman Shoko, Peter Chimwanda, Joice Ndlovu

**Affiliations:** Department of Biology, School of Natural Sciences and Mathematics, Chinhoyi University of Technology, P. Bag 7724 Chinhoyi, Zimbabwe; Department of Mathematics, School of Natural Sciences and Mathematics, Chinhoyi University of Technology, P. Bag 7724, Chinhoyi, Zimbabwe

**Keywords:** *Fadogia ancylantha*, micronutrient deficiency, fermented, non-fermented, supplementation

## Abstract

In this study, the concentrations of the essential elements to the human body N, K, Mg, P, Ca, Fe, Mn, and Zn of the fermented and non-fermented *Fadogia ancylantha* leaf samples were analysed to assess their nutritional value in two different areas in Zimbabwe: Mhangura (Mashonaland West, Province) and Alaska (Mashonaland West Province). Atomic absorption spectroscopy and ultraviolet spectrophotometry techniques were used to measure the concentrations of the minerals. The concentrations of manganese were significantly high (*p* < 0.05) in non-fermented treatments, with Mhangura samples having 0.447 mg/g and Alaska samples having 0.453 mg/g. Iron was high in fermented samples with Mhangura samples having 0.245 mg/g and Alaska samples having 0.270 mg/g. The concentrations of manganese and iron in *Fadogia ancylantha* can be used to supplement the recommended daily doses in pregnant, menstruating, and lactating women. The study, therefore, recommends that *Fadogia ancylantha* be used as a nutraceutical for the supplementation of iron and manganese.

## Graphical abstract

**Figure:**
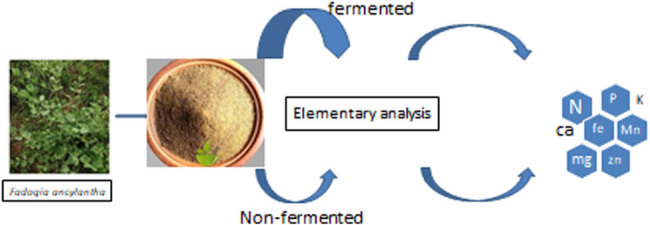

## 1 Introduction

Micronutrient deficiency conditions are a serious health concern globally with statistics indicating that at least two billion people are affected by iron deficiencies, particularly pregnant women and children [1]. These deficiencies have a negative impact on health as they exacerbate the incidences of human immunodeficiency virus (HIV) [2]. Micronutrient deficiencies have prompted the food and medical industries, governments, and other organizations such as the World Health Organization (WHO) and United Nations Children’s Fund (UNICEF) to search for novel food products that contain bioactive compounds and micronutrients that can alleviate these health concerns [3]. In Zimbabwe, women and children less than 5 years old are deficient in the major micronutrients such as iron and zinc, while a large proportion of people living with HIV show micronutrient deficiency [4,5]. Data from WHO show that Zimbabwe has iron and zinc deficiencies ranging from 27 to 64% of the recommended nutrition intake. These statistics have led the Ministry of Health and Child Welfare to adopt the Food Fortification Regulations Statutory Instrument 120 (Fortified Food Regulations), which is meant to increase the micronutrient intake in the population as a way of preventing and controlling deficiency diseases [5]. However, not all Zimbabweans consume fortified products such as mealie-meal, cooking oil, and flour as most people in rural areas and parts of urban areas produce their food products and thus consume them as unfortified [6,7]. Using medicinal teas as dietary supplements is an alternative approach to alleviate nutrient micronutrient deficiencies among population groups that do not consume fortified food products.

Traditionally, medicinal plants are used as dietary supplements and in poly-pharmacology [8]. These plants contain essential elements (iron, manganese, and zinc) and bioactive compounds that are required by the human body for proper day-to-day function and also have a pharmaceutical role [9]. To date, medicinal plants are still being used in the traditional medicinal systems around the world for the treatment and prevention of various ailments in addition to supplementing the diet of pregnant women [10].

Despite the pharmaceutical values of some herbal teas, their metabolites vary depending on the environment where they grow [11]. These variations are a result of ecological factors during their growth [12]. Furthermore, these variations are thought to help plants adapt to their different environments [13] It has been noted that the chemical contents of plants of the same species in different environments may differ significantly [14]. In the traditional systems, physicians assess the quality of medicinal plants by their physical appearance [15]. Therefore, the concept of geo-authentic herbal drugs was established, which stresses the good quality of medicinal plants in particular regions [16].

The leaves of *Fadogia ancylantha,* local ly known as Makoni tea in the commercial market, ha ve become a c ommon herbal tea. The herbal leaves are used to boost the immune system and are used for a variety of other ailments [17]. The local communities prefer the use of *F. ancylantha* over the commercial *Aspalathus linearis* (Rooibos tea) and C *amellia sinensis,* despite several studies that have reported the health benefit of their derived commercial products on non-communicable diseases and micronutrient deficiency conditions [18]. The herbal leaves are used to boost the immune system and treat abdominal pains and a variety of other ailments [17].

Therefore this study investigated the profiles of the essential elements (N, K, Mg, P, Ca, Fe, Mn, and Z) of *F. ancylantha* leaves in two different areas of Zimbabwe (Mhangura and Alaska), and recommended the area with the highest concentrations of these elements to supplement the diet of pregnant women and children.

## 2 Materials and methods

### 2.1 Plant materials

Leaf samples of *F. ancylantha* were collected from Mhangura (latitude −16.88; longitude 30.19) and Alaska (Latitude −17.36; longitude 30.13), which are in Mashonaland West, between May and June 2017 as it is the time local communities harvest the plant for the preparation of the tea. Herbarium samples were collected and identified as *F. ancylantha* at the National Herbarium in Harare, Zimbabwe. Voucher specimens were kept in the herbarium, in the Department of Biology, Chinhoyi University of Technology. The samples were treated in two different ways.

### 2.2 Leaves preparation

Fresh leaves were harvested during the day, and 5 kg of the leaf samples were immediately oven-dried at 40^o^C for 48 h to a moisture content of 8–10% to make the unfermented tea. Another 5 kg of the leaf samples was fermented by spraying the leaves with about 100 ml of distilled water followed by storing the moist leaves in a dark room at room temperature (20–24°C) for 48 h before oven drying at 40°C for 48 h (that is when they would have reached a moisture content of 8–10%), thus making the fermented tea.

### 2.3 Sample preparation and analysis by atomic absorption spectroscopy and ultraviolet spectrophotometry

All leaf samples were then ground into a fine powder using a mortar and pestle followed by storing at 4^o^C. One gram of the powdered plant material from each sample, in triplicate, was placed into appropriately labelled crucibles. Each sample was ashed in an electric muffle furnace (model number STM-6-12) at 500^o^C for 12 h. The ashed samples were then digested in 6 ml of 25% HCl and 6 drops of 55% nitric acid at room temperature, and left to dry. The samples were dissolved in 6 ml of 25% HCl and filtered using a filter paper (Whatman No. 42) into a 50 ml volumetric flask and topped with distilled water to the 50 ml mark. The solutions were analysed for the elements of interest using an atomic absorption spectrophotometer (model number AA320N) with suitable hollow cathode lamps (Table 1). The different elements in these samples were determined by the corresponding standard calibration obtained by using standard grade solutions (0, 10, 50, and 100 ppm) of the elements, i.e. K^+^, Mg^2+^, Ca^2+^, Fe^2+^, Mn^2+^, and Zn^2+^. Phosphorus and boron concentrations were determined using an ultraviolet spectroscope at 450 nm. Nitrogen concentration was determined using the Kjeldahl apparatus where 1 g of the ground leaf sample was weighed, and transferred into the digestion tube which contained 1 g of a catalyst (made of 3.5 g K_2_SO_4_ + 0.0035 g Se) and 12 ml of concentrated sulphuric acid was added. The sample was allowed to digest at 180°C for 2 h and allowed to cool at room temperature. The cool digest was diluted by carefully adding 30 ml of distilled water and was placed into the distillation unit. The distillate was titrated with sodium hydroxide solution (*c* = 0.1 mol/L) to the neutral endpoint. The percentage of nitrogen was determined using the following formula: percentage of nitrogen in sample = (1.4*V* × *N*)/*W*, where *V* is the volume of acid used in the titration (ml), *N* is the normality of the standard acid and *W* is the weight of the sample (g). The experimental design was completely randomized. The mean values of the elements under study were compared using a one-way analysis of variance (ANOVA) and a post hoc test was carried out using the SPSS package.

**Table 1:** The hallowed cathode lamp specifications of the atomic absorption spectroscopy used to quantify the trace elements in *F. ancylantha* herbal tea

Element	K	Ca	Mg	Fe	Mn	Zn	Cu ppm
Wavelength (nm)	266.5	422.7	285.3	279.3	279.3	213.9	324.8
Lamp current (A)	5	5	4	5	5	5	4
Slit width	0.5	0.5	0.5	0.2	0.2	0.1	0.5

## 3 Results

### 3.1 Nitrogen

**Figure 1:**
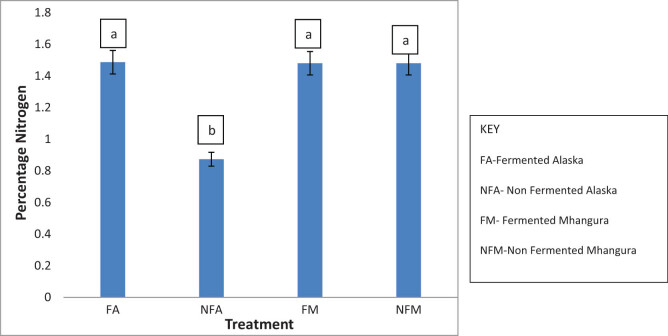
The nitrogen percentage concentrations in different herbal leaf preparations harvested in two different areas of Zimbabwe.

The percentage mean content of nitrogen was high in all treatments except in non-fermented Alaska (Figure 1). Within the fermented group, there were no significant statistical (*p* > 0.05) differences between the tea harvested from Mhangura and Alaska as indicated by the overlapping error bars. In the non-fermented groups, Mhangura had a significantly high (*p* < 0.05) percentage of nitrogen (∼1.48%) while Alaska had a low content of (∼0.87%).

### 3.2 Phosphorous

**Figure 2:**
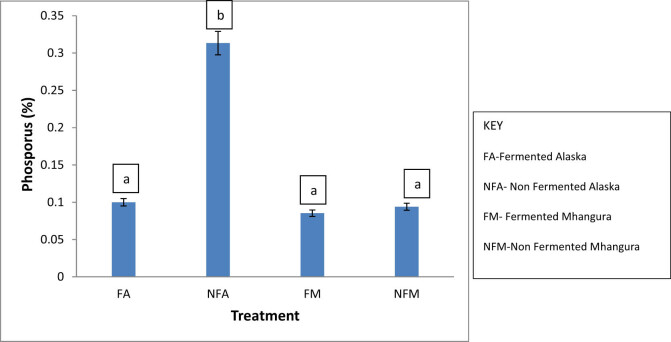
The phosphorus percentage concentrations in different herbal leaf preparations harvested in two different areas of Zimbabwe.

Non-fermented treatments from Alaska had the highest content of 0.3%, which was statistically significant (*p* < 0.05) from the fermented tea from Alaska as indicated by the non-overlap of the error bars. Fermented Alaska, fermented Mhangura, and non-fermented Mhangura tea had lower contents of about 0.1%, which were not statistically significant (*p* > 0.05) from each other as indicated by the overlap of error bars (Figure 2).

### 3.3 Calcium

**Figure 3:**
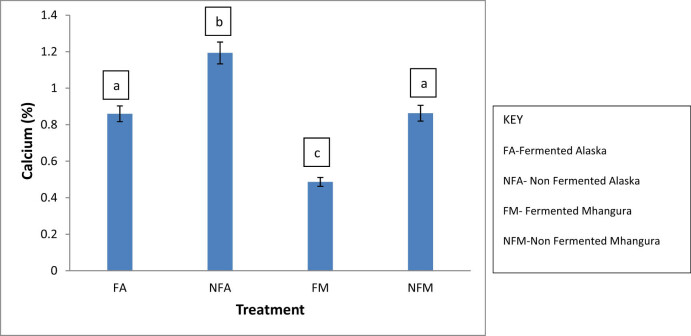
The calcium percentage concentrations in different herbal leaf preparations harvested in two different areas in Zimbabwe.

The highest calcium content was in non-fermented treatments which had contents of about 1 and 2%. Within the tea samples collected from the same location, there was a statistically significant (*p* < 0.05) difference between the fermented and non-fermented tea treatments. The fermented treatments from Mhangura had the lowest (0.5%), and the fermented Alaska and non-fermented Mhangura had almost the same content (0.9%; Figure 3).

### 3.4 Potassium

**Figure 4:**
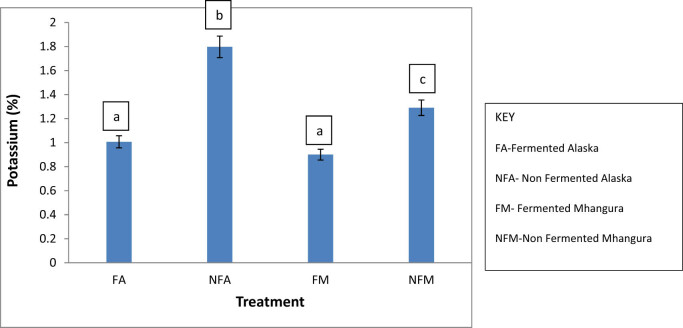
The potassium percentage concentrations in different herbal leaf preparations harvested in two different areas in Zimbabwe.

The Alaska non-fermented treatments had significantly higher (*p* < 0.05) potassium contents of 2%, while the Mhangura non-fermented treatments had significant (*p* < 0.05) lower potassium contents. The potassium content varied with the respective treatments with statistical differences being noted across the treatments (Figure 4).

### 3.5 Magnesium

**Figure 5:**
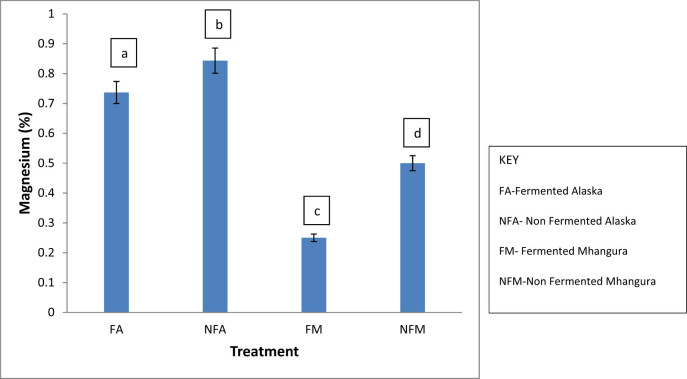
The magnesium percentage concentrations in different herbal leaf preparations harvested in two different areas in Zimbabwe.

The Alaska non-fermented treatments had the highest magnesium content of about 0.9%, while the Mhangura-fermented treatments had the lowest magnesium content of about 0.25% (Figure 5).

### 3.6 Iron

**Figure 6:**
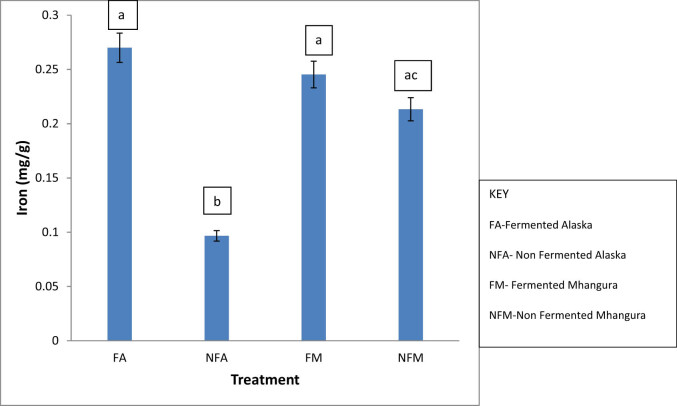
The concentrations of iron (in mg/g) found in different herbal leaf preparations harvested in two different areas in Zimbabwe.

The highest contents of iron are found in fermented treatments in Alaska (0.276 mg/g), while the lowest concentration was noted in the Alaska unfermented treatments (0.098 mg/g). In the non-fermented treatments, Mhangura had the highest of 0.210 mg/g. There was no statistically significant (*p* > 0.05) difference between the iron content in the fermented treatments from Alaska and Mhangura, while statistical differences (*p* < 0.05) were noted in the non-fermented treatments of Alaska and Mhangura as indicated by the non-overlapping error bars (Figure 6).

### 3.7 Manganese

**Figure 7:**
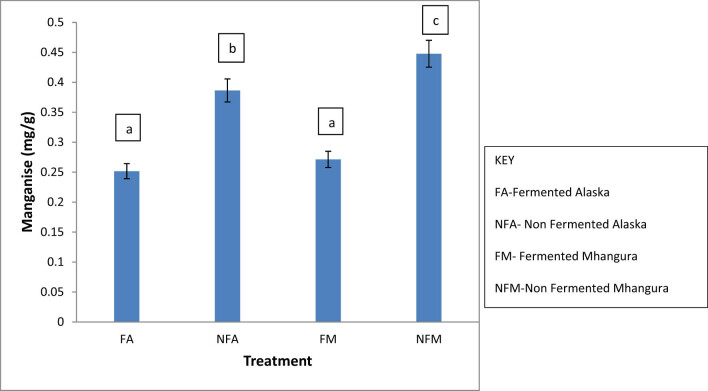
The concentrations of manganese (in mg/g) found in different herbal leaf preparations harvested in two different areas in Zimbabwe.

The manganese content between the treatments was considerably different. The non-fermented treatment from Alaska and Mhangura had significantly higher manganese contents when compared to the fermented treatments from the same locations, with Alaska having a concentration of 0.447 mg/g and Mhangura having a concentration of 0.453 mg/g. The manganese content in the fermented groups was statistically different (*p* < 0.05) within the treatments from the non-fermented treatments (Figure 7).

### 3.8 Zinc

**Figure 8:**
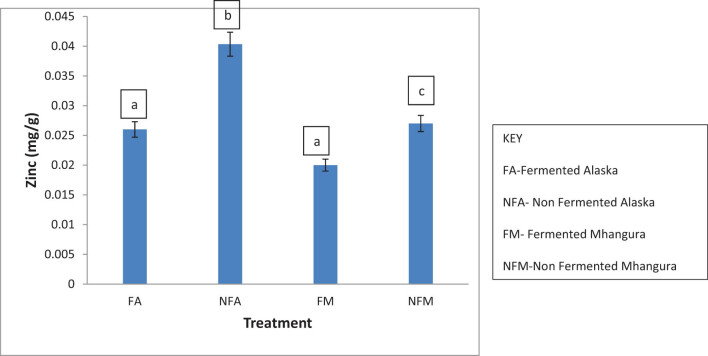
The concentrations of zinc (in mg/g) found in different herbal leaf preparations harvested in two different areas in Zimbabwe.

The mean concentrations of zinc varied from location to location despite the treatment type (Figure 8). Higher concentrations of zinc were found in the non-fermented treatments from Alaska with a concentration of 0.040 mg/g found in Mhangura. The fermented treatments had concentrations ranging from 0.0200 to 0.025 mg/g. There was a statistically significant difference (*p* < 0.05) between the tea harvested in the same location subjected to different treatments.

## 4 Discussion

The consumption of herbal plant materials as nutritional supplements has been recorded in several recent studies, suggesting that herbal plant materials are recognized as nutritionally dense by the nutraceutical industry [19,20,21]. Some plant materials such as leaves provide good sources of non-haeme iron, zinc, manganese, calcium, potassium, and nitrogen which are all elements that are required for normal body functioning [4]. The choice of leaves that are used as ingredients for making teas by the growing herbal tea industry and traditional tea drinkers is influenced by the chemical concentrations of bioactive components such as micro and macro nutrients required for the treatment/prevention of a particular illness or disease [22]. These elements, although present in minute amounts, are essential for the normal development and functioning of the human body [23]. Micronutrients cannot be synthesized by the human body, hence they must be supplied in the normal day-to-day diet [24]. A properly balanced diet should contain the essential macro and micronutrients in quantities that the total supply is adequate to meet demand, as both deficiency and excess can cause a range of nutritional disorders [25]. Therefore, the search for and analysis of different types of new foods can add valuable information about a natural source of micronutrients [26].

The concentration of essential elements in leaves is influenced by two main factors which are the area in which they are harvested and the method used for the preparation of the leaf material being used as an ingredient in making tea [27]. The environmental conditions of an area favour the production of certain bioactive components over others, while the method of preparation has the same effect [28]. In this study, traditional leaves used as a nutraceutical were prepared by subjecting the leaves to two different treatment methods (non-fermentation and fermentation), and also harvested from two different locations was assessed for their elementary composition.

In this study, fermentation significantly increased the iron content of *F. ancylantha* leaves (Figure 6). The high iron content (0.276 mg/g) in fermented leaf samples compared to the non-fermented samples (0.098 mg/g) can be attributed to fermentation. Several studies have shown that fermentation of foods increases the bioavailability of iron [29]. Processing methods like fermentation have been shown to decrease the content of inhibitory phytate which binds to iron and makes it less bioavailable [30]. Furthermore, the difference in the iron content in the tea leaves subjected to the same treatments but from different locations might be attributed to the difference in the environmental conditions in which the tea plant was harvested [31]. A number of studies have postulated that the content of these essential elements in plants is a result of the integrated environmental conditions in which the plant is harvested [32]. Factors such as soil type can also have a positive effect on the bioavailability of iron in plants [33]. In the soil, iron is available as either the soluble ferrous (Fe^2+^) iron or the non-soluble ferric (Fe^3+^) iron [34]. Mhangura soil might have had higher concentrations of ferrous iron (Fe^2+^), which is bioavailable to the plant hence the higher concentration in the sample. On the other hand, the Alaska soils might have had higher concentrations of the non-soluble ferric (Fe^3+^) iron, which was less bio-available to the plant and hence the lower concentrations when compared to the Mhangura leaf samples.

Iron as a component is important in a number of proteins including haemoglobin, myoglobin, erythrocytes, and some enzymes. It is also necessary for growth and development. According to the Recommended Dietary Intakes, to achieve iron balance, adult men need to absorb about 1 mg/day and adult menstruating women about 1.5 mg/day, while pregnant women require more iron, of about 3–3.5 mg/day, to replace the loss due to excretion in urine, feaces, sweat, tears, vaginal fluids and that which is absorbed by the foetus The herbal tea leaves have an average iron content of 0.250 mg/g, which is about one-quarter of the iron required by adult men. The leaves are a good source of iron that can supplement the diet in addition to the other foodstuffs consumed per meal. The high source of iron in the fermented leaves justifies the use of *F. ancylantha* by pregnant women who are prone to iron deficiency. Adequate dietary iron is important for pregnant women as it prevents gestational diabetes mellitus [35]

Manganese is a mineral that the body requires in very minute quantities. This element is stored in the liver, pancreas, kidneys, and bones. It acts as an enzyme cofactor, helping proteins, fats, and carbohydrates to be digested and also breaking free radicals that cause diseases such as cancer and heart disease [36]. Also, in small quantities, this element aids in the production of sex hormones, which help maintain healthy joints and bones [37]. There are other benefits of manganese that include calcium absorption and regulation of diabetes mellitus [38]. It has been noted that individuals suffering from diabetes have lower manganese levels and may need to supplement for the nutrient [39]. The minimum manganese required per day for a pregnant and breastfeeding woman is between 2 and 2.6 mg. The unfermented *F. ancylantha* from Mhangura contains about 0.447 mg/g of manganese and can be used to supplement the trace mineral, especially in people suffering from diabetes, also in pregnant and breastfeeding women in addition to other foodstuff they would have taken during the day.

Zinc, like other trace elements, is also required in very minute amounts in the human body. It plays a major role in the body’s defensive system, as it is an important component in anti-oxidant enzymes such as glutathione and catalase. It also reduces oxidative processes by activating the expression of metallothioneins [40,41]. Among other uses, it is involved in cell division, cell growth, wound healing, and the breakdown of carbohydrates [42]. Zinc is also important in the treatment of diabetes as it is involved in the synthesis, storage, and secretion of insulin [43]. Unfermented Makoni tea from Alaska is a good source, as it contains about 0.040 mg/g of zinc; this in addition to other meals, can help supplement the trace element.

In this study, there was a general increase in iron after the leaves from both locations (Mhangura and Alaska) were subjected to fermentation. It is critical to note that nutraceutical plant materials in the traditional system in Zimbabwe are targeted at certain groups like pregnant women, people suffering from certain diseases, and other target groups. In this case, the use of *F. ancylantha* leaves for iron supplementation for pregnant and menstruating women is justified. The popularization of *F. ancylantha* is critical as pregnant women always have to take supplementary iron during pregnancy. Obtaining iron and other elements like calcium from natural sources may lead to decreased micronutrient deficiency among disadvantaged communities who do not have access to fortified foods as these natural sources are free and easily accessible by people from disadvantaged backgrounds.
